# Presidential Address—Abstract

**Published:** 1898-07

**Authors:** G. V. Brown


### Presidential Address—Abstract.

BY G. V. BROWN.

Address of the Chairman of the Section of Stomatology, delivered before the American Medical Association.

It is my pleasure, as your Chairman, to give the first greeting to the Section of Stomatology of this Association. Stomatologists in fact we have long been, I am sure, but to be so recognized by title is a long step forward in the direction toward which, from year to year, those who have carried the burden of the vast amount of work necessary to this section, have labored, and is certainly worthy of general congratulation. We no longer serve under the somewhat limited, almost ambiguous term of "oral surgeons," but are hereafter to be recognized as those having understanding of the science of the mouth, a tribute complimentary to the scientific character of papers which, published side by side in the Journal of this Association with the essays of the most learned medical and surgical lights of the day, have gained the respect of the other branches, having given those who treat diseases of the mouth their only opportunity to exchange ideas directly with the medical world, the influence of which, in discovering to it the possibilities of applying the technique of the skillful dentist, in conjunction with a knowledge also of surgical procedure and medical science, cannot be over estimated.

The address of your Chairman, Dr. Andrews, last year, in following out the idea of an anniversary celebration, has told the story of the earnest few so completely that reference to the history of the section is unnecessary. It seems, however, that his suggestion as to the necessity for united efforts being made, in the interest of sanitation, in having the mouths of school children regularly examined by capable practitioners of dentistry, and in a general way, instruction given as to the care they ought to receive, is worthy of reiteration, in order that the idea may not escape entirely. An effort made in my own home during the past year toward carrying out the plan has not proven, thus far, of any great practical value, but constant agitation and repeated effort will undoubtedly accomplish much that is at first apparently impossible. There is another line of duty to which I would respectfully call your attention, which also calls for united effort in order that our objects may be furthered.

The essential idea of this section, is that dentists shall also be graduates in medicine, therefore properly entitled to the benefits accruing from association and consultation with physicians. There is a difference of opinion as to the advisability of having the student take the medical degree first, and the special degree in dental surgery afterward, or *vice versa*, but there can be no question as to the importance, and little, if any, of the necessity of having both; but unfortunately for this desideratum the difficulty of accomplishment, owing to lengthening of the required course of instruction for recognition by the National Association of Medical College Faculties, and the National Association of Dental Faculties, while with each it is a mark of progress toward higher education, yet the probable effect, so far as the Section of Stomatology is concerned, and the advance of both professions into that interlying field, fast becoming of value to each, is most unfavorable and bids fair to completely abort, or at least inhibit a promising growth in future usefulness, simply because the number of years of study required is becoming, not only quite beyond the possibility of the average student to attend, but more extensive than the benefits to be derived from the practice of our special branch will warrant, in comparison with other departments of medical science. Therefore the natural result will be that the student of the near future must decide either to be a dentist, pure and simple, without a degree in general medicine, or be obliged to become an oculist, an aurist, a rhinologist, or a practitioner limited to some one or more of the other special fields of medicine, for which he can equip himself with one year, or perhaps a little more, of study, after graduating at some medical college, and even if he take a short post-graduate course abroad, as so many do, will yet have perhaps expended less time and money than would be required to complete his attendance at a reputable dental school.

When this Section was first organized and recognized by the American Medical Association, it was possible to complete the required course for the degree Doctor of Dental Surgery in two years, then get an advanced standing in the medical college and receive a degree Doctor of Medicine in one year afterward. To-day the National Association of Medical Colleges requires four years preparation for graduation, and the Dental Faculty Association three years, with the prospect of an additional year in the near future. It has already become a matter of some difficulty for a graduate of either school to be allowed advanced standing of even one year in the other, so that under the most favorable circumstances it requires five years' study to acquire both degrees at the present time, with every likelihood of the sixth year being added. Thus it seems not altogether improbable that, without proper adjustment of the difficulty, the Section of Stomatology might almost become limited in its active usefulness to the lives of present members.

I believe the good work done by this Section already, and the far reaching benefits resulting from the great advance due to an understanding of the nature and treatment of diseases of the mouth, applied in a scientific manner, by association with other departments of medicine, have established the fact, beyond the peradventure of a doubt, that from a clinical and scientific standpoint, it has earned the right to urge upon each side that an effort be made to further the protection of future influence, by modifying, for the purpose, the exacting requirements upon those who desire to be fully equipped with both degrees.

To take the medical graduate in one year through the necessary technique of operations upon the teeth, in order that he may become an accomplished operator in that short time, is almost impossible. To require students of the dental college to take a full course in anatomy, physiology, chemistry and all the fundamental branches, the same as medical students, has been proven to be, for the average individual, quite too much, in addition to his mechanical instruction, having a tendency to cause neglect of the latter; but in dental schools that are connected with medical colleges and universities, it is always possible for a student, willing to make unusual effort, to take the regular course with the medical student, and at the same time fulfill the requirements of his dental studies and laboratory work. For such, the reward and encouragement should be that, having passed the same examinations, with an average equally as high as his medical brother, he will receive credit for those branches, and be exempt from future examinations, so far as they are concerned, when he undertakes the study of medicine; also, that the dental student so educated shall receive some mark of distinction in his degree, which will serve to note the difference in his favor over other graduates in dentistry, and that the holder of such a degree be allowed to finish his course of medicine in at most two additional years. On the other hand, medical students, at colleges where both branches are taught, may avail themselves of the opportunity to do required work under the instructions in dental operative and prosthetic technics, and having done so be admitted to the senior year of a dental class, with credit allowed them for this work done, even though it had been done while a matriculate in the school of medicine. Such an arrangement would in no wise tend to let down the bars in either profession, and would without doubt encourage many students to make this effort, the enviable result of which would be more fully educated men and a greater number brought up directly in line with the purpose of this Section.

The bibliography of the past year in its valuable additions to science from members of our profession, particularly of this Section, invites discussion in a most alluring manner, were time and your patience sufficient; but in order that all may not escape and because of special interest to myself as bearing upon a subject, the clinical aspect of which has for some time been the object of my investigation, I desire to call attention to the photographs of microscopical sections recently shown by Dr. J. Leon Williams, of London, in line with others by Talbot and statements by Black demonstrating that the blood and nerve supply of the peridental membrane of the tooth is something quite different from that which is depicted in the illustrations of Gray and other anatomical authorities, clearly showing, as they do, nerve filaments and nerve fibres communicating directly with the peridental membrane of the tooth from the main nerve supply, a most important clinical consideration. Last year before this Section, in my paper upon the Hyper Kinesis of the Muscles of Mastication, I endeavored to call attention to the fact that tooth grinding or extreme pressure of the jaws, caused by irritation of the nerve centers controlling those muscles, as a common etiological factor intimately associated with neuralgia of the tri-geminus and other pains of the head, particularly in those forms of the headache which are the accompaniment of neurasthenic conditions. Since the writing of the paper referred to a number of very interesting cases have come under my charge, in which the character of the pain had been mistaken for reflex of uterine diseases, and for which there had been one or more operations performed in the hope of relief, but without success, which in every case was afforded by correction of the source of irritation, discovered to be in the mouth. In one case there was evident a condition such as might be attributed to what is known as eye-strain, and I desire to introduce at this time the expression jaw-strain, as applied to a similar general condition resulting from an irritable weakness of the nerve centers controlling the muscles of mastication and the trigemenal branches directly affected, which without going into more minute explanations, are easily accounted for by an examination of the photo-micrographs referred to. Since through the peridental membrane we have as direct a connection with the main nerve supply as had long been supposed to be through the apical foramin of the pulp of the tooth only, and through tooth grinding could have produced an equivalent condition to that of eye-strain which several writers claim to be the cause of at least fifty percent of all cases of hystero-epilepsy and other disorders of nervous origin.

